# Dupilumab-Induced, Tralokinumab-Induced, and Belantamab Mafodotin–Induced Adverse Ocular Events—Incidence, Etiology, and Management

**DOI:** 10.1097/ICO.0000000000003162

**Published:** 2022-12-15

**Authors:** Tomas Mickevicius, Andrew E. Pink, Maninder Bhogal, David O'Brart, Scott J. Robbie

**Affiliations:** * Department of Ophthalmology, Guy's and St Thomas' NHS Foundation Trust, London, United Kingdom;; † Department of Ophthalmology, Hospital of Lithuanian University of Health Sciences, Kaunas, Lithuania; and; ‡ St John's Institute of Dermatology, Guy's and St Thomas' NHS Foundation Trust, London, United Kingdom.

**Keywords:** dupilumab, tralokinumab, belantamab mafodotin, ocular surface disease

## Abstract

Emerging monoclonal antibody therapies are assuming greater importance in the management of severe and refractory forms of immunity-driven and oncological disorders. However, some have been found to induce adverse ocular events (AOEs) leading to discontinuation of treatment or additional multidisciplinary management. We present the current knowledge concerning AOEs associated with 3 monoclonal antibody therapies: dupilumab, tralokinumab, and belantamab mafodotin. We examine the manifestations of their AOEs, proposed pathophysiological mechanisms, and current treatment recommendations. We identified and reviewed all studies for dupilumab, tralokinumab, and belantamab mafodotin using the keywords “dupilumab,” “tralokinumab,” “belantamab mafodotin,” “conjunctivitis,” and “keratopathy” from January 2016 to November 2021. Conjunctivitis was the most frequently reported AOE in patients with atopic dermatitis receiving dupilumab or tralokinumab. Mild cases were managed with warm compresses for associated meibomian gland dysfunction, artificial tears, and antihistamine/mast cell stabilizer eye drops. In more severe cases, additional anti-inflammatory therapy, with corticosteroid eye drops or ointments, or topical calcineurin inhibitors—such as tacrolimus or ciclosporin—were required. Patients with resistant or refractory multiple myeloma treated with belantamab mafodotin often developed keratopathy, which could necessitate contact lens fitting, or for cycles of belantamab mafodotin to be delayed.

## Abstract 2

Supplemental Digital Content is Available in the Text.

In recent decades, numerous monoclonal antibody therapies have been developed and introduced into modern medical practice. Many are now considered mainstream and effective options in the management of autoimmune disease (eg, rheumatoid arthritis, psoriasis, Crohn disease, and ulcerative colitis^1–3^), atopic conditions (eg atopic dermatitis,^4^ asthma^5^), and cancers (eg, multiple myeloma^6^). However, although the targeted modification of disease pathways by monoclonal antibodies has been shown to positively transform outcomes,^1–6^ these novel pharmaceuticals are capable of driving adverse events that affect a range of major organs and systems,^7^ with data increasingly supporting their potential to exert negative effects on the eye.^8,9^

At present, there are 102 US Food and Drug Administration (FDA)–approved monoclonal antibodies, 41 (40%) of which may be associated with possible ocular side effects (Supplemental Digital Content; Table 1, http://links.lww.com/ICO/B455). In this review, we have focused on 3 recently introduced monoclonal antibody therapeutic interventions that seem to be associated with significant and not infrequent AOEs and which ophthalmologists increasingly need to be aware of and manage actively.^10–13^ Dupilumab, tralokinumab, and belantamab mafodotin are associated with ocular surface adverse events and are of particular interest to those with subspecialist interest in cornea and external eye disease. We examine the manifestations of their AOEs, proposed pathophysiological mechanisms and current treatment recommendations.

## MATERIALS AND METHODS

**FIGURE 1.**
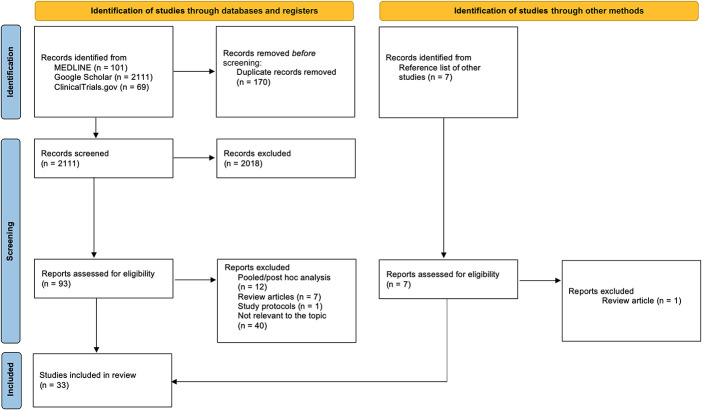
PRISMA 2020 flow diagram. The procedure for literature searching and assessment of eligibility for the review at each stage.

Data retrieval was performed in MEDLINE, Google Scholar, and ClinicalTrials.gov databases. The search strategy for MEDLINE was ((“dupilumab"[Supplementary Concept] OR “dupilumab"[All Fields]) AND “conjuntivitis"[All Fields]) AND ((humans[Filter]) AND (2016[pdat]) AND (english[Filter])), ((“tralokinumab"[Supplementary Concept] OR “tralokinumab"[All Fields]) AND (“conjunctivities"[All Fields] OR “conjunctivitis"[MeSH Terms] OR “conjunctivitis"[All Fields] OR “conjunctivitides"[All Fields])) AND ((humans[Filter]) AND (2016[pdat]) AND (english[Filter])) and (“belantamab"[All Fields] AND (“keratopathies"[All Fields] OR “keratopathy"[All Fields])) AND ((humans[Filter]) AND (2016[pdat]) AND (english[Filter])). For Google Scholar, search was performed using “Dupilumab AND conjunctivitis”, “Tralokinumab AND conjunctivitis”, and “Belantamab mafodotin AND keratopathy” terms. Records were screened and assessed for eligibility following PRISMA 2020 flow diagram (Fig. 1). We restricted our search to human studies published in English from January 2016 to November 2021. Two thousand one hundred, eleven publications were gathered after a search in the databases and additional 7 publications were found after reviewing reference lists. After removing duplicates, reading study titles, their abstracts, and full-text articles, 33 studies were included in our review: 24 randomized clinical trials (RCTs) and 9 non-RCT publications (case series and reports), which discuss clinical findings of dupilumab-induced, tralokinumab-induced, and belantamab mafodotin–induced adverse ocular events (AOEs). Publications from the reference lists of the analyzed articles were also considered as a valid source of information.

## RESULTS

Table 1 shows the key study characteristics and main clinical findings of RCTs included in the review. It summarizes randomization processes and the incidence of specific monoclonal antibody treatment-related AOEs found in each study. Findings are reported in greater detail below:

**TABLE 1.** Study Characteristics and Main Clinical Findings of RCTs Included in the Review

Authors	RCT Phase	Monoclonal Antibody	Primary Indication	Safety Evaluation Period	Number of Study Participants	Groups	Main Clinical Findings
Blauvelt et al.^16^	2	DP	Atopic dermatitis	32 w	178	DP qw P qw	The incidence of treatment-related conjunctivitis: 8.2% with DP versus 0% with P;
Simpson et al.^17^	3	DP	Atopic dermatitis	16 w	671 (SOLO1) 708 (SOLO2)	DP qw DP q2w P qw	The incidence of treatment-related “unspecified conjunctivitis” (SOLO1): 3% with DP qw versus 5% DP q2w versus 1% with P; The incidence of treatment-related “unspecified conjunctivitis” (SOLO2): 4% with DP qw versus 4% DP q2w versus <1% with P; The incidence of treatment-related allergic conjunctivitis (SOLO1): 3% with DP qw versus 5% DP q2w versus 1% with P; The incidence of treatment-related “allergic conjunctivitis” (SOLO2): 1% with DP qw versus 1% DP q2w versus 1% with P
Worm et al.^18^	3	DP	Atopic dermatitis	36 w	422	DP qw or q2w DP q4w DP q8w P	The incidence of any treatment-related conjunctivitis (bacterial, viral, allergic conjunctivitis, atopic keratoconjunctivitis): 5.4% with DP qw or q2w versus 4.6% DP q4w versus 3.6% DP q8w versus 4.9% with P; The incidence of treatment-related “allergic conjunctivitis”: 0% with DP qw or q2w versus 2.3% DP q4w versus 0% DP q8w versus 0% with P
Blauvelt et al.^19^	3	DP	Atopic dermatitis	52 w	623	DP qw + TCS DP q2w + TCS P + TCS	The incidence of any treatment-related conjunctivitis (bacterial, viral, allergic conjunctivitis, atopic keratoconjunctivitis): 32% with DP qw + TCS versus 31% DP q2w + TCS versus 15% with P + TCS
Bruin-Weller et al.^20^	3	DP	Atopic dermatitis	28 w	325	DP qw + TCS DP q2w + TCS P + TCS	The incidence of any treatment-related conjunctivitis (bacterial, viral, allergic conjunctivitis): 16.4% with DP qw + TCS versus 28% DP q2w + TCS versus 11.1% with P + TCS
Zhao et al.^21^	3	DP	Atopic dermatitis	16 w	165	DP q2w P q2w	The incidence of treatment-related conjunctivitis: 10% with DP q2w versus 5% with P q2w
Simpson et al.^22^	3	DP	Atopic dermatitis	16 w	251	DP q2w DP q4w P q2w	The incidence of treatment-related conjunctivitis: 9.8% with DP q2w versus 10.8% DP q4w versus 4.7% with P q2w
Cork et al.^23^	3	DP	Atopic dermatitis	52 w	78	DP 2 mg kg ^−1^ qw DP 4 mg kg ^−1^ qw	The incidence of treatment-related conjunctivitis: 18% with DP 2 mg kg ^−1^ qw versus 16% with DP 4 mg kg ^−1^ qw
Cork et al.^24^	3	DP	Atopic dermatitis	52 w	38	DP 2 mg kg ^−1^ qw DP 4 mg kg ^−1^ qw	The incidence of treatment-related conjunctivitis: 12% with DP 2 mg kg ^−1^ qw versus 31% with DP 4 mg kg ^−1^ qw
Paller et al.^25^	3	DP	Atopic dermatitis	16 w	362	DP (300 mg) q4w + TCS DP (100 or 200 mg) q2w + TCS P + TCS	The incidence of treatment-related conjunctivitis (baseline weight <30 kg): 20.6% with DP (100 mg) q2w + TCS versus 6.7% with DP (300 mg) q4w + TCS versus 3.3% with P + TCS; The incidence of treatment-related conjunctivitis (baseline weight = or >30 kg): 8.5% with DP (200 mg) q2w + TCS versus 6.7% with DP (300 mg) q4w + TCS versus 5% with P + TCS; The incidence of treatment-related conjunctivitis (overall): 14.8% with DP (100/200 mg) q2w + TCS versus 6.7% with DP (300 mg) q4w + TCS versus 4.2% with P + TCS; The incidence of treatment-related keratitis (baseline weight <30 kg): 1.6% with DP (100 mg) q2w + TCS versus 0% with DP (300 mg) q4w + TCS versus 0% with P + TCS; The incidence of treatment-related keratitis (baseline weight = or >30 kg): 0% with DP (200 mg) q2w + TCS versus 0% with DP (300 mg) q4w + TCS versus 0% with P + TCS; The incidence of treatment-related keratitis (overall): 0.8% with DP (100/200 mg) q2w + TCS versus 0% with DP (300 mg) q4w + TCS versus 0% with P + TCS
Castro et al.^44^	3	DP	Asthma	52 w	1902	DP (200 mg) q2w DP (300 mg) q2w P (1.14 mL) q2w P (2.00 mL) q2w	The incidence of conjunctivitis: 2.3%% with DP q2w (combined) versus 3.3% with P q2w (combined)
Wenzel et al.^46^	2	DP	Asthma	40 w	769	DP (300 mg) q2w, q4w DP (200 mg) q2w, q4w P	No cases of AOEs reported
Rabe et al.^47^	3	DP	Asthma	36 w	210	DP q2w P q2w	No cases of AOEs reported
Bachert et al.^45^	3	DP	Chronic sinusitis	52 w	276 (LIBERTY NP SINUS–24) 448 (LIBERTY NP SINUS–52)	DP q2w (LIBERTY NP SINUS–24) P q2w (LIBERTY NP SINUS–24) DP q2w (LIBERTY NP SINUS–52) DP q2w to q4w (LIBERTY NP SINUS–52) P q2w (LIBERTY NP SINUS–52)	The incidence of conjunctivitis (LIBERTY NP SINUS–24 and LIBERTY NP SINUS–52): 1.6% with DP (combined) versus 0.4% with P q2w
Bachert et al.^51^	2	DP	Chronic sinusitis	52 w	60	DP qw P qw	No cases of AOEs reported
Wollenberg et al.^28^	2	TR	Atopic dermatitis	12 w	204	TR (45 mg) q2w TR (150 mg) q2w TR (300 mg) q2w P q2w	The incidence of treatment-related conjunctivitis: 2% with TR (45 mg) q2w versus 5.9% with TR (150 mg) q2w versus 3.9% with P q2w
Wollenberg et al.^52^	3	TR	Atopic dermatitis	16 w	802 (ECZTRA 1) 794 (ECZTRA 2)	TR qw P qw	The incidence of treatment-related conjunctivitis (ECZTRA 1): 7.1% with TR qw versus 2% with P qw; The incidence of treatment-related conjunctivitis (ECZTRA 1): 0.2% with TR qw versus 0% with P qw; The incidence of treatment-related conjunctivitis (ECZTRA 2): 3% with TR qw versus 1.5% with P qw; The incidence of treatment-related conjunctivitis (ECZTRA 2): 0.3% with TR qw versus 0% with P qw
Silverberg et al.^29^	3	TR	Atopic dermatitis	32 w	380	TR q2w + TCS P q2w + TCS	The incidence of treatment-related conjunctivitis (initial 16-week treatment period): 13.1% with TR q2w + TCS versus 5.6% with P q2w + TCS; The incidence of treatment-related conjunctivitis (continuation treatment period—responders): 4.3% with TR q2w + TCS versus 1.4% with TR q4w + TCS versus 2.4% with P q2w + TCS The incidence of treatment-related conjunctivitis (continuation treatment period—nonresponders): 4.2% with TR q2w + TCS versus 7.6% with P q2w + TCS
Gutermuth et al.^30^	3	TR	Atopic dermatitis	26 w	275	TR q2w + TCS P q2w + TCS	The incidence of treatment-related conjunctivitis: 9.4% with TR q2w + TCS versus 4.4% with P q2w + TCS; The incidence of treatment-related keratoconjunctivitis: 0.7% with TR q2w + TCS versus 0% with P q2w + TCS; The incidence of treatment-related keratitis: 0.7% with TR q2w + TCS versus 0.7% with P q2w + TCS
Panettieri et al.^49^	3	TR	Asthma	72 w	1207 (STRATOS 1) 856 (STRATOS 2)	TR q2w or q4w (STRATOS 1) P q2w or q4w (STRATOS 1) TR q2w (STRATOS 2) P q2w (STRATOS 2)	No cases of AOEs reported
Busse et al.^50^	3	TR	Asthma	54 w	140	TR q2w P q2w	No cases of AOEs reported
Russel et al.^48^	2	TR	Asthma	26 w	79	TR q2w P q2w	No cases of AOEs reported
Trudel et al.^35^	1	BM	Multiple myeloma	Up to 42 w	38 (part 1) 35 (part 2)	BM (0.03–4.6 mg kg ^−1^) (Part 1) BM 3.4 mg kg ^−1^ (Part 2)	Adverse corneal events (Part 2): 91% Grade 1 or Grade 2 versus 9% Grade 3
Lonial et al.^36^	2	BM	Multiple myeloma	Up to 48 w	196	BM 2.5 mg kg ^−1^ q3w -BM 3.44 mg kg ^−1^ q3w	The incidence of Grade 1–2 keratopathy: 43% with BM 2.5 mg kg ^−1^ q3w versus 54% with BM 3.44 mg kg ^−1^ q3w; The incidence of grade 3 keratopathy: 27% with BM 2.5 mg kg ^−1^ q3w versus 20% with BM 3.44 mg kg ^−1^ q3w; The incidence of Grade 4 keratopathy: 0% with BM 2.5 mg kg ^−1^ q3w versus 1% with BM 3.44 mg kg ^−1^ q3w

AOEs, adverse ocular events; BM, belantamab mafodotin; DP, dupilumab; P, placebo; q2w, every 2 weeks; q3w, every 3 weeks; q4w, every 4 weeks; q8w, every 8 weeks; qw, every week; TCS, topical corticosteroids; TR, tralokinumab; w, weeks.

### Dupilumab-Associated Adverse Ocular Events Reported by RCTs

Dupilumab (Dupixent, Regeneron Pharmaceuticals Inc.) is a human monoclonal IgG4 antibody licensed for use in the treatment of moderate-to-severe atopic dermatitis (AD) (subcutaneous administration, 300 mg every 2 weeks) and moderate–severe asthma. It inhibits IL-4 and IL-13 signal transduction by binding to the IL-4 receptor alpha (IL-4Rα), a subunit of both IL-4 and IL-13 receptors.^14^ This results in the downregulation of markers of epidermal proliferation, inflammatory mediators, upregulation of structural proteins, lipid metabolism proteins, and epidermal barrier proteins resulting in the normalization of skin.^15^

A phase 2 trial (LIBERTY AD EVALUATE; ClinicalTrials.gov identifier: NCT02210780)^16^ examined whether a weekly dose of subcutaneous dupilumab (300 mg) affects a response of antibodies to tetanus toxoid with reduced diphtheria toxoid and acellular pertussis (Tdap) and quadrivalent meningococcal polysaccharide vaccines in adults with moderate-to-severe AD, as well as its efficacy and safety profiles. After 32 weeks of follow-up, only in the dupilumab group study participants developed mild or moderate conjunctivitis.

The LIBERTY AD SOLO1 and SOLO2 were both randomized, double-masked, placebo-controlled, parallel-group phase 3 studies.^17^ They evaluated efficacy and safety profiles of subcutaneous dupilumab (300 mg) in adults with moderate-to-severe AD, whose disease was inadequately controlled by topical treatment. Over the 16-week treatment period, the rates of treatment-related “unspecified conjunctivitis” in SOLO2 was higher in both dupilumab groups compared with placebo but were relatively low in general. SOLO1 showed that “allergic conjunctivitis” was also more prevalent in both dupilumab groups, but the rate of it in SOLO2 study was the same in all the groups (all 1%). There was only 1 case of ophthalmic herpes simplex infection in one of the dupilumab groups and 1 case of herpes zoster ophthalmicus infection in both, placebo and dupilumab groups (all <1%).

After the latter RCTs concluded, some of the patients were recruited to the new LIBERTY SOLO-CONTINUE^18^ study. At the end of the treatment period, safety analysis revealed that “allergic conjunctivitis” was present only in the dupilumab group (2.3%), where it was given to the study participants once a month. Furthermore, other types of conjunctivitis, including “atopic keratoconjunctivitis”, were most common in the group where dupilumab was administered to study participants once every 7 or 14 days (5.4%).

LIBERTY AD CHRONOS^19^ was a phase 3 study where patients were randomly assigned to 2 subcutaneous dupilumab (300 mg) groups or placebo. Topical cutaneous corticosteroids (TCS) were given additionally to all study participants with or without topical calcineurin inhibitors. The results showed that over the 13-month treatment period, the incidence of treatment-related bacterial, viral, “allergic” conjunctivitis, and “atopic keratoconjunctivitis” was higher in the “dupilumab plus TCS” groups than in the “placebo plus TCS” group. Most of the cases were mild or moderate. There were only 2 study participants (1%) who received dupilumab once a week plus TCS and 1 study participant (<1%) who received placebo plus TCS who had “severe” conjunctivitis. One patient in the former group decided to discontinue the treatment after developing “atopic keratoconjunctivitis” in one of his eyes.

A similar trial was published by de Bruin-Weller et al in 2018 (LIBERTY AD CAFÉ ClinicalTrials.gov Identifier: NCT02755649).^20^ Their results showed that in study participants with moderate-to-severe AD, “treatment-emergent conjunctivitis” was the most prevalent in those who received “subcutaneous dupilumab (300 mg) plus TCS” once every 2 weeks (28%) after a 28-week safety follow-up. The incidence rate was lower in patients who received it only once every week (16.4%) or placebo (11.1%).

In the most recently published double-masked, placebo-controlled, parallel-group phase 3 study (ClinicalTrials.gov registration: NCT03912259),^21^ patients with moderate-to-severe AD received subcutaneous dupilumab (300 mg) or matching placebo for a total of 16-week treatment period. Safety profile analysis revealed a 2 times higher frequency of treatment-related “conjunctivitis” in patients who received dupilumab, compared with those who received placebo.

The LIBERTY AD ADOL^22^ clinical trial investigated the efficacy and safety of subcutaneous dupilumab in adolescent moderate-to-severe AD patients. Only patients who were older than age 12 years, but younger than 18 years, were eligible to enroll in this study. The results showed that the most frequent AOE among adolescents was “conjunctivitis” after a 4-month follow-up. Incidence was higher in the dupilumab groups (9.8% and 10.8%) compared with the placebo group (4.7%). None of these effects were severe or led to the discontinuation of the treatment.

The same age group and population of study participants was chosen in the phase 3 LIBERTY AD PED-OLE^23^ (pediatric open-label extension) RCT. The most common AOE in the study was also found to be “conjunctivitis”. Over the 52-week treatment period, the incidence rate in the “dupilumab 2 mg kg^−1^ qw” and “dupilumab 4 mg kg^−1^ qw” groups reached 18% and 16%, respectively. None of these cases were serious, and all of them resolved by the end of treatment.

Cork et al published a two-part sequential study^24^ where the efficacy and safety of subcutaneous dupilumab was explored in an even younger population. This study involved children aged 6 years or older to younger than 12 years with uncontrolled, severe AD. In this phase 2a study, Part A patients received single-dose subcutaneous dupilumab (2 or 4 mg kg^−1^), with an 8-week follow-up. Short-term safety assessments performed subsequently showed only 1 patient of 19 (5%) in the 4 mg kg^−1^ group developing treatment-related “conjunctivitis.” Part B study participants received weekly doses of subcutaneous dupilumab (2 or 4 mg kg^−1^) for an additional 4 weeks with further 8-week follow-up. At the end of both parts of the study, 2 of 19 (11%) patients had developed treatment-related “conjunctivitis”; in the treatment period, there were no patients in the 2 mg kg^−1^ group who developed any AOEs. In the sequential phase 3 open-label extension study, patients continued to receive their assigned treatment regimen once every week for 13 months. Safety analysis performed over this treatment period showed a substantially higher incidence of treatment-related “conjunctivitis” in the 4 mg kg^−1^ group than in the 2 mg kg^−1^ group; none of which were severe or led to discontinuation of the treatment.

Paller et al^25^ published a double-masked, placebo-controlled phase 3 trial, which evaluated the efficacy and safety profile of dupilumab when administered to children with severe AD aged 6 to 11 years, who received dermal TCS. Safety assessment of different regimes revealed that the most common AOE in the studied population was “conjunctivitis” over a 16-week treatment period. It was more prevalent in children who received dupilumab compared with placebo. One patient of 63 (1.6%) in the dupilumab 100 mg q2w ± TCS group developed keratitis, and 1 child of 59 (1.7%) in the dupilumab 200 mg q2w ± TCS group had to discontinue treatment because of severe bacterial conjunctivitis.

### Tralokinumab-Associated Adverse Ocular Events Reported by RCTs

Tralokinumab (Adtralza, LEO Pharma A/S) is another human monoclonal IgG4 antibody licensed for use in the treatment of moderate-to-severe AD.^26^ It competitively blocks the binding of IL-13 to IL-13 Rα1 and IL-13 Rα2, restricting the connection with the IL-13 receptor and subsequent downstream signaling.^27^

A phase 2b study (NCT02347176)^28^ revealed the highest incidence of treatment-related “conjunctivitis” in the 150 mg tralokinumab group (5.9%) and the lowest in the 45 mg tralokinumab group (2%).

ECZTRA 1 and ECZTRA 2^29^ were both randomized, double-masked, placebo-controlled RCTs. Treatment-emergent “conjunctivitis” was found to be the most frequent (ECZTRA 1) or the second most common (ECZTRA 2) AOE, which was more predominant in the tralokinumab groups compared with the placebo groups. Only subjects receiving tralokinumab were diagnosed with “keratoconjunctivitis” in the course of the study.

AOEs were investigated in another double-masked, placebo-controlled phase 3 trial (ECZTRA 3).^29^ The safety analysis after initial 16-week treatment period showed that treatment-related “conjunctivitis” was associated more frequently with tralokinumab (n = 33; 13.1%) than with placebo (n = 7; 5.6%). All AOEs were mild or moderate, and most recovered by the end of the treatment. However, 1 patient had to discontinue subcutaneous tralokinumab because of conjunctivitis. After the initial treatment period, a group of patients who responded to tralokinumab were rerandomized into smaller groups in a 1:1 ratio to receive tralokinumab every 2 or 4 weeks. A placebo group was rerandomized simultaneously, where nonresponders started to receive 300 mg subcutaneous tralokinumab every 2 weeks plus dermal TCS, as needed, for an additional 16 weeks. The safety assessment results during the course of this continuation treatment period revealed that “conjunctivitis” was more frequent among nonresponders to placebo (7.6%) or the initial treatment with tralokinumab (4.2%), compared with responders, who continued to receive placebo (4.9%) or were subdivided to receive tralokinumab every 4 weeks (1.4%).

In a 26-week, multicenter, double-masked, placebo-controlled, phase 3 trial published by Gutermuth et al (ECZTRA7),^30^ the efficacy and safety profiles of subcutaneous tralokinumab (300 mg) in adults with severe ciclosporin-refractory AD were evaluated. Safety analysis of 275 patients showed a higher frequency of conjunctivitis among patients receiving monoclonal antibody therapy compared with placebo. One patient of 137 (0.7%) in the former group developed “keratoconjunctivitis”. All AOEs were mild or moderate and resolved by the end of the treatment. None of them led to treatment discontinuation.

### Clinical Presentations of Dupilumab-Associated Ocular Surface Disease

**FIGURE 2.**
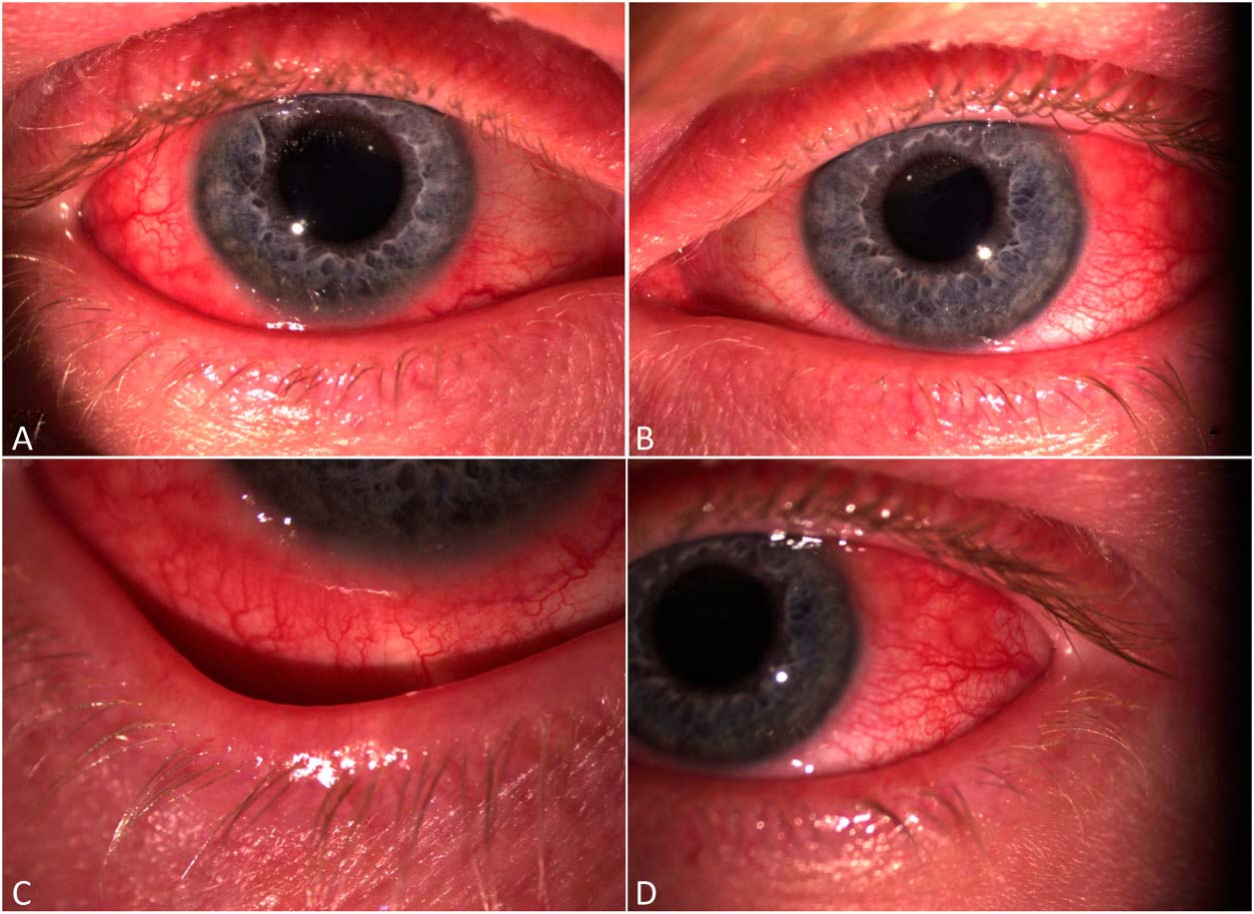
Slitlamp photographs of dupilumab-associated conjunctivitis in a 21-year-old man commenced on fortnightly dupilumab for atopic dermatitis 2 years previously. Images show diffuse bulbar conjunctival hyperemia affecting the right (A) and left (B) eyes, with inferior limbitis on the right eye (C) and marked inflammation temporally in the left eye (D).

Popiela et al^10^ reviewed clinical presentations of 9 patients of 28 (32.1%) with severe AD who developed “dupilumab-associated conjunctivitis” (DAC). All these patients presented with either bilateral papillary (66.7%) or bilateral follicular (33.3%) reactions. Two patients developed limbal nodules, and there were 4 cases of cicatrizing ectropion, punctal stenosis, and periocular dermatitis. The study included 2 sisters who presented with conjunctival cicatrization after starting treatment with dupilumab, one of them developing a progressive disease and biopsy-confirmed precancerous actinic keratosis with dysplasia. Calabrese et al^31^ published a case series which reviewed 14 patients with a moderate-to-severe form of AD affected by DAC, despite preventative therapy with hyaluronic acid eye drops which were prescribed for them at the start of treatment. Interestingly, bilateral ocular involvement was detected in only 3 patients (21.4%), while in 11 patients (75.6%), only 1 eye was affected; all 14 cases had peripalpebral eczema. Recently, a potential side effect of uveitis arising from dupilumab therapy has been reported.^32,33^ Figure 2 shows a clinical case of dupilumab-associated ocular surface disease from our department in a 21-year-old man.

### Belantamab Mafodotin–Associated Adverse Ocular Events Reported by RCTs

Belantamab mafodotin (Belamaf, GlaxoSmithKline) is a humanized IgG1 antibody–drug conjugate, which targets B-cell maturation antigen and is used in the treatment of multiple myeloma (MM).^34^

A phase 1 study, BMA117159/DREAMM-1,^35^ was the first to explore the safety and tolerability of belantamab mafodotin in patients with refractory or relapsed MM. It consisted of dose escalation (1) and dose-expansion (2) parts. In Part 1, corneal events occurred in 53% of study participants. In the dose-expansion part, corneal events occurred even more frequently—at 63%. However, most of these effects were considered mild.

In an open-label, two-arm, phase 2 study DREAMM-2,^36^ adult patients with relapsed or refractory MM, with disease progression after three or more lines of therapy, were recruited to evaluate the efficacy and safety of belantamab mafodotin, introduced by intravenous infusion every 3 weeks (until disease progression or unacceptable toxicity). The most common AOE in this study was keratopathy. For most of the study participants, it was mild or moderate—in 41 of 95 (43%) study participants in the 2.5 mg/kg belantamab mafodotin group and in 53 of 99 (54%) study participants in the 3.4 mg/kg belantamab mafodotin group. Nonetheless, there were also a considerable number of severe cases of keratopathy (27% and 20% in the 2.5 mg/kg and 3.4 mg/kg groups, respectively). Keratopathy was also the most common reason for treatment discontinuation. The belantamab mafodotin dose had to be reduced in 49 study participants (22 of 95 [23%] patients and 27 of 99 [27%] patients). In 93 of 194 participants, administration had to be delayed (45 of 95 [47%] patients and 48 of 99 [48%] patients) because of changes in the cornea. The most common patient-reported ocular symptoms were blurred vision and dry eye.

### Clinical Presentations of Belantamab Mafodotin–Associated Ocular Surface Disease

**FIGURE 3.**
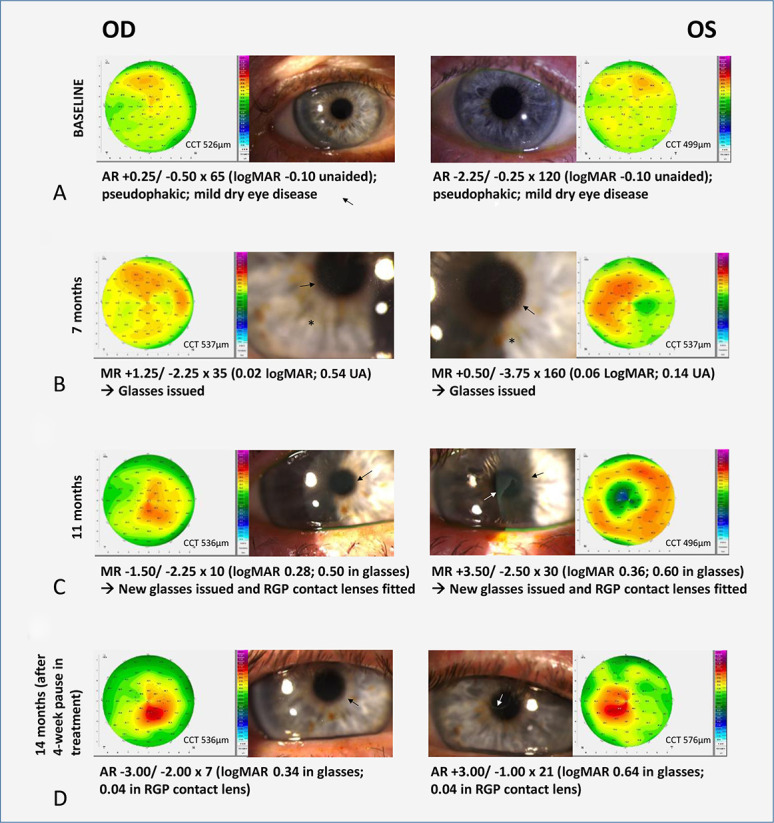
Slitlamp photographs and Scheimpflug topographic maps in a 74-year-old man commenced on belantamab 3-weekly for multiple myeloma. A, Baseline images. B, Corneal epithelial microcysts (black arrows) evident 2 months after starting treatment, with irregularity evident on the topography maps. C, 11 months after commencing treatment, denser microcystic changes with additional whorls, sheet-like and linear opacities (white arrows), and increasing irregularity on Scheimpflug imaging, with central flattening in the left eye (inducing a hypermetropic shift). D, At 14 months, after 2 months of cessation of treatment, there appeared to be some reversal of epithelial changes and associated improvement in topographic shape. MR, manifest refraction; RGP, rigid gas permeable; UA, unaided.

Only limited data have been released on belantamab mafodotin–related structural corneal changes in patients with refractory or resistant MM. Bilateral microcystic-like epithelial changes (MECs) in the corneal periphery, which later tend to migrate to the central corneal area, are the most commonly reported findings.^13,37,38^ Figure 3 shows the changes that can occur in the corneal epithelium over several months after commencement of therapy, with associated corneal irregularity—demonstrable on tomographic imaging—driving changes in refraction that may vary considerably over several weeks. These changes may necessitate frequent changes in spectacle correction and even rigid gas permeable contact lens fitting. The corneal side effects tend to develop faster in those patients receiving a higher dose of belantamab mafodotin.^13^ Anterior segment optical coherent tomography (AS-OCT)^37,39^ or in vivo confocal microscopy^37,40^ corneal scans usually permit the identification of hyper-reflective lesions in corneal epithelium, which are believed to significantly improve after a 5-week drug-free period. Epithelial changes appear to be concentrated in the paracentral cornea, with relative sparing of the center, which may be demonstrable on AS-OCT^39^ or corneal topography (see Fig. 3C, left eye, where a hypermetropic shift resulted). *Bausell et al*^13^ found that once the treatment is restarted, subsequent reappearance of MECs tends to be more rapid and takes longer to regress after each interruption of the treatment.

## DISCUSSION

### The Incidence of Dupilumab-Associated and Tralokinumab-Associated Ocular Surface Disease

The reviewed phase 2 and 3 RCTs report an increased rate of AOEs, especially conjunctivitis, in patients with atopic dermatitis receiving dupilumab or tralokinumab when compared with placebo (Table 1). Incidence rates ranged from 3.6% to 31% and from 2% to 13.1%, respectively. They were mostly mild or moderate and resolved on discontinuation of treatment.^14–22,25–27^ There are only 3 cases reported where patients have had to stop treatment because of severe AOEs (1 receiving dupilumab developed severe atopic keratoconjuntivitis^18^ and 2 receiving tralokinumab developed severe conjunctivitis^27,28^). The frequency of AOEs quoted in RCTs for dupilumab has generally been lower than those generated from postmarket surveillance and case series, indicating a need for continued vigilance in determining the rates and severity of AOEs in relation to monoclonal antibody therapies. Halling et al^41^ published a meta-analysis of 22 observational studies where the efficacy and safety of dupilumab in the treatment of AD was evaluated. In total, data from 3303 AD patients were gathered. The most frequently reported side effect during the treatment with dupilumab was found to be conjunctivitis, which was reported by 26.1% of patients AD. The pooled proportion was higher in the studies published in Europe and Asia, compared with those published in North America (30.8%, 36.4%, and 11.2%, respectively). In a prospective 1-year observational cohort study of their first 100 patients treated with dupilumab, Sears et al^12^ found that most AEs were eye-related, with 63% reporting some form of conjunctivitis or dry eye symptoms.

### Mechanism of Dupilumab-Associated and Tralokinumab-Associated Adverse Ocular Events

The exact pathophysiological mechanisms of dupilumab-induced or tralokinumab-induced AOEs are not fully understood. Some studies^42,43^ suggest that by binding to IL-4Rα and, therefore, inhibiting IL-4 and IL-13, these monoclonal antibodies prevent conjunctival goblet cells from being activated, thereby inducing hypoplasia and decreased mucin production, with a consequent effect on the stability of the tear film and the function of the mucosal epithelial barrier. Bakker et al^42^ found a significant scarcity of conjunctival goblet cells, along with T-cell and eosinophilic infiltrates, after evaluating diagnostic conjunctival biopsies of the inferior fornix from AD patients with DAC. Lack of mucin is believed to lead to conjunctivitis by causing increased irritation of the ocular surface.^42,43^

### Dupilumab-Associated and Tralokinumab-Associated Adverse Ocular Events in Other Type 2 Immunity-Driven Diseases

Interestingly, rates of conjunctivitis in patients receiving subcutaneous dupilumab for other type 2 immunity-driven diseases seem to be lower, which may indicate that pathways specific to AD are at play in driving the side effect. In a randomized, double-masked, placebo-controlled, parallel-group, phase 3 trial LIBERTY ASTHMA QUEST,^44^ no clinically significant difference was found between the incidence rates of conjunctivitis among patients with uncontrolled moderate-to-severe asthma receiving monoclonal antibody therapy (2.3%) compared with those in a placebo group (3.3%). The LIBERTY NP SINUS-24 and LIBERTY NP SINUS-52^45^ RCT found 7 of 440 (1.59%) patients, with severe chronic rhinosinusitis with nasal polyps, developed conjunctivitis in comparison with 1 of 282 patients (0.35%) in the placebo group. Other published RCTs^46–51^ report no cases of any AOEs, after conducting safety analysis among patients with severe asthma or chronic rhinosinusitis who received dupilumab or tralokinumab during the established treatment period. Because early trial data indicate that tralokinumab may be less likely to drive AOEs compared with dupilumab, for the same indications, a careful examination of real-world evidence will be necessary to establish this and whether IL-4, IL-13, or both are the drivers for the conjunctivitis observed.^26–28,48–50,52^

### Risk Factors of Dupilumab-Associated Ocular Surface Disease

Some studies have reported possible factors that may increase the risk of developing AOEs in patients with moderate-to-severe AD receiving monoclonal antibodies. According to the available data, these patients tend to be older,^53^ more likely to be male,^31,53^ have more advanced disease^31,53,54^ for a longer period of time,^53^ and have other atopic comorbidities.^53,54^ Sears et al^12^ identified that over half of those reporting conjunctivitis after starting dupilumab had a documented history of prior allergic conjunctivitis.

### Management of Dupilumab-Associated Ocular Surface Disease

There are currently no standard guidelines to help clinicians diagnose and treat monoclonal antibody therapy–induced AOEs. Nonetheless, some management recommendations exist. In cases of DAC, it is considered important to anticipate the side effect, ensure close communication between ophthalmologists and prescribing dermatologists, and to determine the severity of the disorder.^55^ Many patients have preexisting allergic eye disease and meibomian gland dysfunction.^56^ Mild conjunctivitis can be managed by applying warm compresses, using artificial tears and antihistamine/mast cell stabilizer eye drops. These may be prescribed before the initial ophthalmic evaluation. In cases of moderate-to-severe conjunctivitis, additional anti-inflammatory therapy is recommended by prescribing eye drops or ointments containing corticosteroids (eg fluorometholone 0.1%–1%) and calcineurin inhibitors (eg tacrolimus 0.03%–0.1% or ciclosporin); liberal application of topical tacrolimus to the lids and lid margins seems to be particularly effective.^31,57–59^ Severe cases may require intensive, high-potency, topical corticosteroid treatment (dexamethasone 0.1%^60–62^ or prednisolone acetate 1%^63,64^), ideally preservative-free if available. Clinicians should be alert to the possibilities of comorbidities or alternative diagnoses (particularly in patients with unilateral red eye or visual loss) because conditions such as herpes simplex keratitis and keratoconus occur more frequently in patients with atopic disease.^56^ Reducing the frequency of dupilumab to monthly rather than fortnightly may also ameliorate side effects.^62^ The long-term effects of the moderate severity conjunctivitis and limbitis observed in some patients remain unknown.

### The Incidence of Belantamab Mafodotin–Associated Ocular Surface Disease

Belantamab mafodotin is the latest drug approved by the US FDA in cases of refractory or resistant MM. The phase 1 clinical trial investigating this humanized monoclonal antibody reported that 63% of study participants developed corneal events, of which 9% were severe.^43^ From a clinical perspective, 77% of cases were found to have superficial punctate keratitis, variably associated with epithelial microcysts (63%), corneal stromal edema (14%), or corneal opacities (23%). The median time to onset of these corneal events was 23 days. A subsequent phase 2 clinical trial revealed that keratopathy was both most common AOE and reason for treatment discontinuation.^34^ The results of this study also showed that the median time to resolution after treatment exposure was 21 – 63.5 days, depending on the given dose. Permanent loss of vision due to belantamab mafodotin–induced AOEs was not reported in either of the study groups.

### Mechanism of Belantamab Mafodotin–Associated Adverse Ocular Events

The mechanism behind belantamab mafodotin–induced corneal changes is unknown. Farooq et al^65^ proposed that particles of belantamab mafodotin could reach the cornea through the tear film or the limbus and may be internalized by the basal corneal epithelium cells through the process of macropinocytosis and activate apoptosis. It is believed that belantamab mafodotin–containing corneal epithelium cells in various stages of apoptosis continue to migrate centrally and anteriorly, while the cells that have completed apoptosis are shed. However, new epithelial cells are generated at the limbus, which are believed to replace belantamab mafodotin–containing cells that have undergone apoptosis, when the treatment is delayed or discontinued.

### Management of Belantamab Mafodotin–Associated Ocular Surface Disease

In the first instance, alternative causes for any corneal changes should be considered—these may include corneal dystrophy, dry eye disease, infectious keratitis, other drug-induced corneal changes, and autoimmune disease-related keratopathy. In particular, given the context of MM, paraproteinaemic keratopathy, which may affect any layer of the cornea, should be considered among the differentials.^66^

A keratopathy and visual acuity scale was developed by hematologists/oncologists and ophthalmologists to provide some guidance on the management of belantamab mafodotin–induced AOEs.^43,67^ Side effects are graded based on severity into 4 different levels (grade 1—mild, grade 2—moderate, and grade 3 and 4—severe). Each of them requires a different clinical approach and dose modification. In case of grade 1 events, treatment with belantamab mafodotin should be continued at the same initial dose without any interruption. In the presence of grade 2 to 4 corneal events, it is advised that a dose be delayed until there is an improvement to grade 1 or the side effect has resolved completely and only then consider restarting the treatment at a lower initial dose. Moreover, if grade 4 corneal events develop, it is recommended that permanent treatment discontinuation be considered when symptoms worsen despite appropriate management.

Supportive measures, including updating spectacle correction and fitting of contact lenses (including rigid gas permeable contact lenses), may facilitate continuation of therapy. To date, corneal epithelial delamination has not been described in this context, but it is conceivable that this could permit a patient to continue on the drug under circumstances where a favorable response to a pause in belantamab mafodotin therapy was not observed.

## CONCLUSION

Treatment with the monoclonal antibodies dupilumab, tralokinumab, and belantamab mafodotin is associated with an increased incidence of AOEs, principally conjunctivitis and superficial keratopathies. In most cases, these effects are mild, but they may affect tolerance of therapies that are often third-line and indicated for severe disease—consequently, they may drive discontinuation of therapy under circumstances where alternatives are limited or might pose a higher risk of systemic side effects. Clinical observation will be critical to determining rates of AOEs arising from monoclonal antibody therapies, the pathways that drive them and their effective management.

## Supplementary Material

SUPPLEMENTARY MATERIAL
