# Supplementary material for: Dupilumab-Induced, Tralokinumab-Induced, and Belantamab Mafodotin–Induced Adverse Ocular Events—Incidence, Etiology, and Management
Source: Cornea. 2022 Dec 15;42(4):507–19. doi: 10.1097/ICO.0000000000003162 (PMC9973444; doi:10.1097/ICO.0000000000003162)
Supplement: SUPPLEMENTARY MATERIAL [file cornea-42-507-s001.docx]

**Supplemental Digital Content. Table 1.** Ocular side effects of FDA-approved monoclonal antibodies^1,2^.

| **Monoclonal Antibody** | **Brand name** | **Target** | **Primary indication** | **U.S. FDA**  **approval**  **year** | **Ocular side effects** |
| --- | --- | --- | --- | --- | --- |
| Infliximab | Remicade | TNF | Crohn disease | 1998 | Optic neuritis; unexplained visual loss; |
| Trastuzumab | Herceptin | HER2 | Breast cancer | 1998 | Corneal erosion; corneal lesion; focal lesion of limbal conjunctiva; |
| Adalimumab | Humira | TNF | Rheumatoid arthritis | 2002 | Optic neuritis; |
| Cetuximab | Erbitux | EGFR | Colorectal cancer | 2004 | Blepharitis; cicatricial ectropion; conjunctivitis; keratitis; eyelid madarosis; trichomegaly; |
| Bevacizumab | Avastin | VEGF | Colorectal cancer | 2004 | Blurred vision; chemosis of conjunctiva; corneal edema; endophthalmitis; floaters in visual field; functional visual loss; keratitis; macular hole; macular infarction; retinal tear; toxic anterior segment syndrome |
| Natalizumab | Tysabri | a4 integrin | Multiple sclerosis | 2004 | Acute retinal necrosis; progressive outer retinal necrosis; |
| Panitumumab | Vectibix | EGFR | Colorectal cancer | 2006 | Conjunctivitis; excessive tear production; eye irritation; eyelash formation, growth; hyperemia of eye; keratitis; perforation of cornea; Trichomegaly |
| Ranibizumab | Lucentis | VEGF | Macular degeneration | 2006 | Blepharitis; cataract; conjunctival edema; conjunctival hemorrhage; conjunctival hyperemia; corneal abrasion; corneal edema; dry eye syndrome; endophthalmitis; eye irritation; foreign body sensation, in the eyes; hypotony of eye; injection site hemorrhage; conjunctival erosion; conjunctival retraction; iritis; leaking filtering bleb; pain in eye; raised intraocular pressure; reduced visual acuity; retinal detachment; retinal pigment epithelial detachment with tear of retinal pigment epithelium; secondary cataract; vitreous detachment; vitreous floaters; vitreous hemorrhage; |
| Certolizumab pegol | Cimzia | TNF | Crohn disease | 2008 | Demyelinating disease of central nervous system; oculomotor nerve demyelination; optic neuritis; retinal hemorrhage; uveitis; |
| Ustekinumab | Stelara | IL-12/23 | Psoriasis | 2009 | Herpes zoster with ophthalmic complication |
| Golimumab | Simponi | TNF | Rheumatoid and psoriatic arthritis, ankylosing spondylitis | 2009 | Optic neuritis |
| Tocilizumab | RoActemra, Actemra | IL-6R | Rheumatoid arthritis | 2010 | Nodular scleritis |
| Denosumab | Prolia | RANK-L | Bone Loss | 2010 | Bilateral cataracts |
| Ipilimumab | Yervoy | CTLA-4 | Metastatic melanoma | 2011 | Blepharitis; blurred vision; choroidal retinal neovascularization; episcleritis; iritis; myasthenia gravis, ocular; ophthalmoplegia; orbital myositis; panuveitis; scleritis; sudden visual loss; uveitis; Vogt-Koyanagi-Harada disease |
| Ado-trastuzumab emtansine | Kadcyla | HER2 | Breast cancer | 2012 | Blurred vision; conjunctivitis; corneal lesion; dry eyes; epiphora |
| Nivolumab | Opdivo | PD1 | Melanoma, non-small cell lung cancer | 2014 | Blurred vision; iridocyclitis; iritis; myasthenia gravis, ocular; panuveitis; unexplained visual loss; uveitis; Vogt-Koyanagi-Harada disease |
| Pembrolizumab | Keytruda | PD1 | Melanoma | 2014 | Corneal epithelial defect; decreased intraocular pressure; functional visual loss; iritis; myasthenia gravis, ocular; optic neuritis; uveitis; |
| Alemtuzumab | Lemtrada; MabCampath, Campath-1H | CD52 | Multiple sclerosis; chronic myeloid leukemia | 2014 | Disorder of optic nerve; retinal pigment epithelial abnormality; thyroid eye disease |
| Necitumumab | Portrazza | EGFR | Non-small cell lung cancer | 2015 | Conjunctivitis |
| Dinutuximab | Qarziba; Unituxin | GD2 | Neuroblastoma | 2015 | Blurred vision; photophobia; mydriasis; fixed or unequal pupils; optic nerve disorder; eyelid ptosis, and papilledema; neuroblastoma |
| Alirocumab | Praluent | PCSK9 | High cholesterol | 2015 | Cataract |
| Elotuzumab | Empliciti | SLAMF7 | Multiple myeloma | 2015 | Cataract |
| Ixekizumab | Taltz | IL-17a | Psoriasis | 2016 | Conjunctivitis |
| Atezolizumab | Tecentriq | PD-L1 | Bladder cancer | 2016 | Iritis; myasthenia gravis, ocular; uveitis; visual impairment; Vogt-Koyanagi-Harada disease |
| Brodalumab | Siliq, LUMICEF | IL-17R | Plaque psoriasis | 2017 | Conjunctivitis |
| Dupilumab | Dupixent | IL-4R α | Atopic dermatitis | 2017 | Blepharitis; conjunctivitis; dry eyes; itching of eye; keratitis |
| Avelumab | Bavencio | PD-L1 | Merkel cell carcinoma | 2017 | Hemolytic anemia; iritis; myasthenia gravis, ocular; uveitis; visual impairment; Vogt-Koyanagi-Harada disease |
| Durvalumab | IMFINZI | PD-L1 | Bladder cancer | 2017 | Iritis; myasthenia gravis, ocular; uveitis; visual impairment |
| Mogamulizumab | Poteligeo | CCR4 | Mycosis fungoides or Sézary syndrome | 2018 | Conjunctivitis |
| Cemiplimab | Libtayo | PD-1 | Cutaneous squamous cell carcinoma | 2018 | Iritis; uveitis; visual impairment; Vogt-Koyanagi-Harada disease |
| Moxetumomab pasudotox | Lumoxiti | CD22 | Hairy cell leukemia | 2018 | Bilateral cataracts; blurred vision; dry eyes; pain in eye; periorbital edema |
| Polatuzumab vedotin | Polivy | CD79b | Diffuse large B-cell lymphoma | 2019 | Blurred vision |
| Brolucizumab | Beovu | VEGF-A | Neovascular age-related macular degeneration | 2019 | Blurred vision; cataract; conjunctival hemorrhage; conjunctivitis; corneal abrasion; endophthalmitis; pain in eye; raised intraocular pressure; retinal detachment; retinal hemorrhage; retinal pigment epithelial tear; retinal vascular occlusion; retinal vasculitis; uveitis; vitreous detachment; vitreous floaters |
| Enfortumab vedotin | Padcev | Nectin-4 | Urothelial cancer | 2019 | Blurred vision; keratitis; blurred vision; limbal stem cell deficiency; dry eyes |
| fam-trastuzumab deruxtecan | Enhertu | HER2 | HER2+ metastatic breast cancer | 2019 | Dry eyes |
| Belantamab mafodotin | BLENREP | B-cell maturation antigen | Multiple myeloma | 2020 | Blurred vision; dry eyes; keratitis; photophobia; |
| Naxitamab | DANYELZA | GD2 | High-risk neuroblastoma and refractory osteomedullary disease | 2020 | Unequal pupils; blurred vision; visual impairment; accommodation disorder; photophobia; mydriasis |
| Dostarlimab | Jemperli | PD-1 | Endometrial cancer | 2021 | Iridocyclitis; iritis; unexplained visual loss; uveitis; Vogt-Koyanagi-Harada disease |
| Amivantamab | Rybrevant | EGFR, cMET | NSCLC w/ EGFR exon 20 insertion mutations | 2021 | Keratitis; uveitis |
| Tralokinumab | Adtralza | IL-13 | Atopic dermatitis | 2021 | Blepharitis; conjunctivitis; dry eyes; itching of eye; keratitis; |
| Tisotumab vedotin | TIVDAK | Tissue factor | Cervical cancer | 2021 | Blepharitis; conjunctivitis; dry eyes; keratitis; |

**TNF** - Tumour Necrosis Factor; **HER2** - Human Epidermal Growth Factor Receptor 2; **EGFR** - Estimated Glomerular Filtration Rate; **VEGF** - Vascular Endothelial Growth Factor; **IL** – Interleukin; **CTLA-4** - Cytotoxic T-Lymphocyte Associated Protein 4; **PD1** - Programmed Cell Death Protein 1; **CD52** - Cluster of Differentiation 52; **GD2** – Ganglioside 2; **PCSK9** - Proprotein convertase subtilisin/kexin type 9; **CCR4** - C-C Motif Chemokine Receptor 4; **cMET** - mesenchymal-epidermal transition (cMET) tyrosine kinase receptor;

**REFERENCES**

**1.** Reichert JM. Antibody therapeutics approved or in regulatory review in the EU or US [The Antibody Society web site]. Available at: https://www.antibodysociety.org/resources/approved-antibodies. Accessed January 17, 2022.

**2. IBM MICROMEDEX [database online].** IBM Watson Health**;**
